# Serotonin after β-Adrenoreceptors’ Exposition: New Approaches for Personalized Data in Breast Cancer Cells

**DOI:** 10.3390/jpm11100954

**Published:** 2021-09-25

**Authors:** Ana Salomé Correia, Diana Duarte, Isabel Silva, Henrique Reguengo, José Carlos Oliveira, Nuno Vale

**Affiliations:** 1 OncoPharma Research Group, Center for Health Technology and Services Research (CINTESIS), Rua Dr. Plácido da Costa, 4200-450 Porto, Portugal; anncorr07@gmail.com (A.S.C.); dianaduarte29@gmail.com (D.D.); 2 Institute of Biomedical Sciences Abel Salazar (ICBAS), University of Porto, Rua de Jorge Viterbo Ferreira, 228, 4050-313 Porto, Portugal; 3 Faculty of Pharmacy, University of Porto, Rua de Jorge Viterbo Ferreira, 228, 4050-313 Porto, Portugal; 4 Clinical Chemistry, Department of Laboratory Pathology, Centro Hospitalar Universitário do Porto (CHUP), Largo Prof. Abel Salazar, 4099-313 Porto, Portugal; isa_camp_sil@hotmail.com (I.S.); hlreguengo@gmail.com (H.R.); director.sqc@chporto.min-saude.pt (J.C.O.); 5 Unit for Multidisciplinary Research in Biomedicine (UMIB), University of Porto, Rua de Jorge Viterbo Ferreira, 228, 4050-313 Porto, Portugal; 6 Department of Community Medicine, Information and Health Decision Sciences (MEDCIDS), Faculty of Medicine, University of Porto, Al. Prof. Hernâni Monteiro, 4200-319 Porto, Portugal

**Keywords:** serotonin, MCF-7 cells, propranolol, isoprenaline, ICI 118,551

## Abstract

Serotonin is an important monoamine in the human body, playing crucial roles, such as a neurotransmitter in the central nervous system. Previously, our group reported that β-adrenergic drugs (ICI 118,551, isoprenaline, and propranolol) influence the proliferation of breast cancer cells (MCF-7 cells) and their inherent production of adrenaline. Thus, we aimed to investigate the production of serotonin in MCF-7 cells, clarifying if there is a relationship between this production and the viability of the cells. To address this question, briefly, we treated the MCF-7 cells with ICI 118,551, isoprenaline, and propranolol, and evaluated cellular viability and serotonin production by using MTT, Sulforhodamine B (SRB) and Neutral Red (NR) assays, and HPLC-ECD analysis, respectively. Our results demonstrate that isoprenaline promotes the most pronounced endogenous synthesis of serotonin, about 3.5-fold greater than control cells. Propranolol treatment also increased the synthesis of serotonin (when compared to control). On the other hand, treatment with the drug ICI 118,551 promoted a lower endogenous synthesis of serotonin, about 1.1-fold less than what was observed in the control. Together, these results reveal that MCF-7 cells can produce serotonin, and the drugs propranolol, isoprenaline and ICI 118,551 influence this endogenous production. For the first time, after modulation of the β-adrenergic system, a pronounced cellular growth can be related to higher consumption of serotonin by the cells, resulting in decreased levels of serotonin in cell media, indicative of the importance of serotonin in the growth of MCF-7 cells.

## 1. Introduction

**Figure 1:**
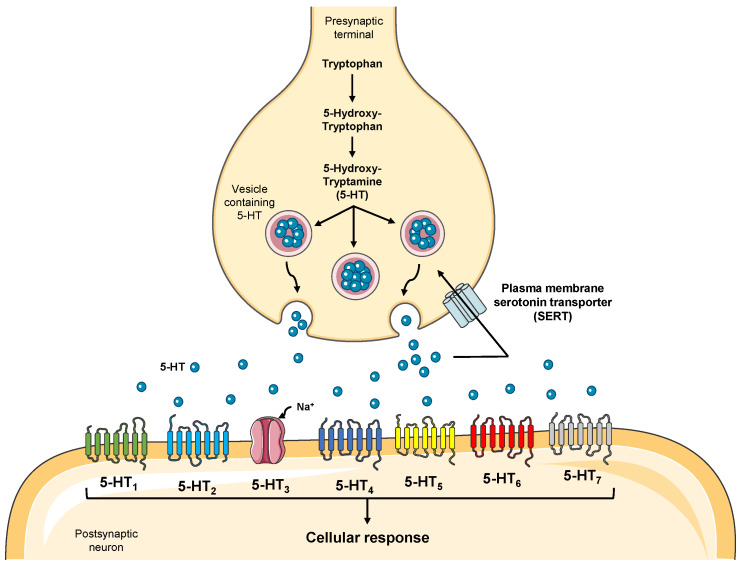
Representation of serotonin production by the CNS and major serotonin receptor families. 5-HT is produced at the presynaptic end of neurons and is originated from the metabolism of tryptophan. This neurotransmitter is transported into vesicles that travel to the synaptic cleft, where they release this monoamine. 5-HT may interact with seven families of receptors (5HT_1–7_) or can be reuptake by the SERT transporter. 5-HT receptors are mostly G protein-coupled, except for the 5-HT_3_ receptor, which is a ligand-gated ion channel. The binding of this monoamine can trigger a wide range of cellular responses [2].

Serotonin (5-Hydroxytryptamine, 5-HT) is a monoamine that plays a crucial and well-defined role as a neurotransmitter in the central nervous system (CNS), as a local mediator in gastrointestinal tract motility, and as a vasoactive in the blood [1], acting through seven families of 5-HT receptors (5-HT_1–7_), known to be distributed throughout the various organs of the human body (Figure 1), comprising a total of 13 subtypes (5-HT_1A_, 5-HT_1B_, 5-HT_1D_, 5-HT_1E_, 5-HT_1F_, 5-HT_2A_, 5-HT_2B_, 5-HT_2C_, 5-HT_3_, 5-HT_4_, 5-HT_5A_, 5-HT_6_, and 5-HT_7_) [2]. 5-HT is produced by the metabolism of the amino acid tryptophan, being synthesized through a pathway that starts in a process in which L-tryptophan is converted into 5-hydroxy-L-tryptophan by an enzyme named tryptophan hydroxylase (TPH). Then, this compound is converted to 5-HT by an aromatic L-amino acid decarboxylase [3,4].

**Figure 2:**
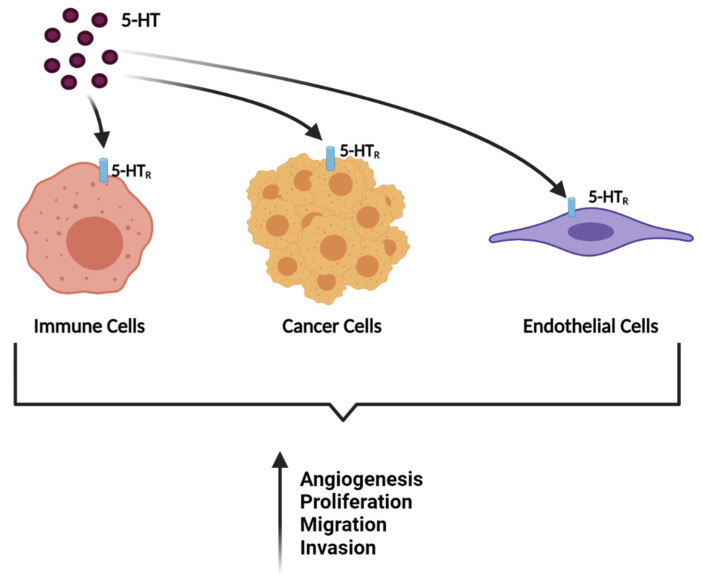
Through its receptors (5-HT_R_), 5-HT interacts with cancer, immune, and endothelial cells, inducing several processes such as proliferation, migration, and angiogenesis. Adapted from [5]. Created with BioRender.com. Available online: http://biorender.com/ (accessed on 17 August 2021).

Recently, several studies have demonstrated a relationship between different types of cancer and signalling pathways that are induced by neurotransmitters. In addition to this relationship observed in cancer cells, it is also observed in immune and endothelial cells that comprise the tumour microenvironment [5]. Indeed, strong evidence has emerged on the role of 5-HT in the progression of various types of cancer. Namely, several studies describe the presence of specific 5-HT receptors in cancer cells. This indicates the involvement of this monoamine in important tumorigenesis processes, such as tumour growth and its ability to invade and metastasize. Thus, 5-HT can be classified as a strong mitogenic factor for cancer and non-cancer cells that comprise the tumour microenvironment (Figure 2) [6,7].

Several in vitro studies have shown that this growth stimulatory effect happens in different cancer cell lines, such as prostate [8], bladder [9], colorectal [10], and breast [11]. In most studies, the effect of serotonin on tumour growth usually involves the 5-HT_1_ and 5-HT_2_ receptors, but this involvement depends on the type of cancer [2]. Being breast cancer one of the most common cancer types worldwide [12], it is important to understand which factors may underline the progression of this alarming disease. Indeed, in vitro studies reveal an important role of 5-HT in this type of cancer. For example, recently, it was demonstrated that 5-HT plays important role in the metabolism and proliferation of breast cancer cells by interfering with different signalling pathways through 5-HT2A/C receptors. These signalling pathways are Jak1/STAT3, which upregulates the process of glycolysis, and adenylyl cyclase/PKA, responsible for increasing mitochondrial biogenesis [13]. Concentration-dependent growth was also found when these cells were stimulated with increasing concentrations of serotonin and DOI (2,5-dimethoxy-4-iodoamphetamine hydrochloride), an agonist of 5-HT2 receptors. Additionally, during tumour progression, the expression of various proteins was also evaluated, and an increased expression of the TPH enzyme was observed. There was also a change in expression of the different receptor subtypes, and cell signalling mediated by these receptors also changed, which resulted in increased proliferation of these cells and increased resistance to apoptosis [14].

Recently, our group reported that three drugs that interact with the adrenergic system (ICI 118,551, isoprenaline, and propranolol; Table 1) influence the proliferation of breast cells (tumorigenic MCF-7 cells) and their ability to produce adrenaline. It was observed that these cells have the potential to synthesize and release adrenaline, influenced by the exposure to agonists/antagonists of β-adrenoreceptors [15]. Indeed, evidence from the literature suggests the presence of β-adrenoreceptors in cancer cells, important for different processes of initiation and progression of the disease [16]. With special relevance, in MCF-7 breast cancer cells, it is known that β2-adrenoreceptor is expressed, contributing to the malignant phenotype [17].

Hereupon, our group aimed to investigate whether there is de novo production of serotonin in MCF-7 cells, as well as to clarify whether there is a relationship between this production and cell viability, after the application of the above-mentioned drugs.

Despite strong evidence of 5-HT involvement in tumour progression, no study has yet reported serotonin quantification directly in MCF-7 cells after administration of β-adrenergic drugs. Usually, this evidence is assessed by the presence of 5-HT receptors or by the measure of TPH1 protein expression, which is related to 5-HT production. This paper describes, for the first time, an analytical methodology that allows quantitative measurement of 5-HT in MCF-7 cells, after the application of drugs that interact with the adrenergic system (ICI 118,551, isoprenaline and propranolol). It is intended to be a tool for future studies on the role of 5-HT in the progression of breast cancer and other types of cancer.

## 2. Materials and Methods

### 2.1. Materials

Dulbecco’s Modified Eagle’s Medium (DMEM), Fetal Bovine Serum (FBS), and Penicillin-Streptomycin mixture were obtained from Millipore Sigma (Merck KGaA, Darmstadt, Germany). Bisbenzimide H33342 trihydrochloride (Hoechst 33342; cat. no. B2261), Thiazolyl Blue Tetrazolium Bromide (MTT; cat. no. M5655), Neutral Red Solution (cat. no. N2889), Sulforhodamine B (cat. no. S1402), propranolol (cat. no. P0884), and isoprenaline (cat. no. I5627) were purchased from Sigma-Aldrich (Merck KGaA, Darmstadt, Germany). paclitaxel (Taxol; cat. no. 1097) and ICI 118,551 (cat. no. 0821) were purchased from Tocris Bioscience (Bristol, UK). Waters Alliance 2695 pump, 3030 Reagent kit^®^, Rheodyne loop injector, and the Decade electrochemical detector (ECD) were purchased from, respectively, Waters Corporation (Milford, MA, USA), Chromsystems GmbH (Munich, Germany), and Antec Scientific (Zoeterwoude, The Netherlands).

### 2.2. Cell Culture

Human breast cancer cell line MCF-7 (American Type Culture Collection, Virginia, USA) was incubated at a temperature of 37 °C, in a humidified atmosphere (95% of air, 5% of CO_2_). These cells were cultivated in DMEM, supplemented with 10% of heat-inactivated FBS and 1% of penicillin/streptomycin (1000 U/mL; 10 mg/mL), according to the recommendations of the American Type Culture Collection. For their maintenance, cells were cultured in a monolayer, being subcultured once a week, when a confluence of 75–80% was reached. The cell media was renewed every 3 days. Before each experiment, cells were trypsinized (0.25% trypsin-EDTA), centrifuged (1100 rpm, 5 min; Hettich, Tuttlingen, Germany), and seeded at an optimal density of 2.3 × 10^4^ cells/mL (obtained through cell growth curve assay; Section 3.1) in 96-well plates, having an attachment period of 24 h.

### 2.3. Cell Growth Curve Assay

After being trypsinized and centrifuged, cells were seeded in 96-well plates at an initial density of 1.0 × 10^4^ cells/cm^2^. Then, cells were incubated at a temperature of 37 °C, in a humidified atmosphere (95% of air and 5% of CO_2_) for 24h. After this period, Hoechst 33342 (1 mg/mL in DMSO) was diluted in culture medium to a value of 5 μg/mL. Then, this solution was added to each well (200 μL), in a period of incubation of 10 min, protected from light. After that, images representing each well were taken using an automated microscope (Lionheart™ FX, BioteK Instruments, Winooski, VT, USA) configured with DAPI light cube. Total cell number was calculated using the software Gen5.0 (version 4), based on Hoechst staining. This procedure was repeated every 24 h for 148 h. Cell media were removed every 3 days. Cells were routinely checked for mycoplasma contamination.

### 2.4. Cell Treatment

Paclitaxel was dissolved in dimethylsulfoxide (DMSO) and applied to the cells at concentrations of [0.1–500 nM]. Isoprenaline, ICI 118,551 and propranolol were dissolved in distilled water and applied to the cells at concentrations of, respectively, [0.01–10 µM], [0.01–1 µM] and [1–100 µM]. In the case of the treatment of the cells with paclitaxel, controls were composed of DMSO 0.1% *v*/*v*. Values of cellular viability, respective concentration-response curves, and half-maximal inhibitory concentration (IC_50_) values for each drug, after 24 h of treatment, were determined by performing MTT (3-(4,5-dimethylthiazol-2-yl) -2,5-diphenyltetrazolium bromide) tetrazolium reduction assay, NR uptake protocol, and SRB assay. Values of serotonin production, in the case of the treatment of the cells with isoprenaline (10 µM), propranolol (100 µM) and ICI 118,551 (1 µM), were quantified by HPLC-ECD assay, after 24 h of treatment.

### 2.5. Cell Viability Assays

Cell viability assays were performed using the MTT assay, NR uptake protocol, and SRB assay. After being attached to 96-well plates, paclitaxel, isoprenaline, propranolol or ICI 118,551 were added to the cells in different concentrations, for a period of 24 h. After this procedure, the cells were exposed to distinct viability assays. For the MTT assay, the cell culture medium was removed, and the MTT solution (0.5 mg/mL in PBS; 100 μL/well) was added to the cells, followed by an incubation period of 3 h, protected from the light. Finally, this solution was aspirated, 100 µL of DMSO was added to each well and the plate was placed on a plate shaker for 10 min. Absorbance values at 570 nm were determined in the automated microplate reader (Tecan Infinite M200, Switzerland). To perform the NR uptake assay, the NR medium (1:100 in culture medium) was prepared the day before and, before being added to the cells, this solution was centrifugated (10 min, 1800 rpm), to remove any precipitated dye crystals. Then, the cell medium was discarded, and 100 µL of NR medium was added to each well, followed by an incubation period of 2 h, protected from light. After this period, this solution was aspirated, washed with PBS (200 µL), and 200 µL of NR Destain Solution (50% of 96% ethanol, 49% deionized water, 1% glacial acetic acid) was added to each well of the plate. Finally, the plate was subjected to a slight agitation for 10 min, and the absorbance at 540 nm was determined in the automated microplate reader. For the SRB assay, the culture medium was removed, and the cells were washed with PBS (400 µL). Then, the cells were fixed with cold 10% trichloroacetic acid (200 µL/well) for 30 min. After that, these cells were stained with SRB solution for 1 h (200 µL/well; room temperature). The dye that exceeded was discarded by rinsing with tap water and, finally, the dye that was bound to the proteins was dissolved with 400 µL of Tris-NaOH solution (10 mM). Absorbance values at 540 nm were determined in the automated microplate reader.

### 2.6. Cell Morphology Visualization

After each treatment condition, the cell morphology was assessed. Leica DMI6000 B Automated Microscope (Wetzlar, Germany) was used to observe and capture MCF-7 cells treated with paclitaxel, propranolol, ICI 118,551, and isoprenaline.

### 2.7. HPLC-ECD

After being plated, attached, and being in contact with propranolol (100 µM), ICI 118,551 (1 µM), and isoprenaline (10 µM) for 24 h, cells and their respective supernatant were divided into different fractions, and perchloric acid 2M (1:10) was added. Then, the samples were subjected to filtration processes (SpinX filters, Costar), centrifugation (12,000× *g*, 5 min), and freezing processes (−20 °C) before analysis. Finally, using the 3030 Reagent kit for HPLC analysis of serotonin in the urine, the analysis of serotonin was carried out, following the specific recommendations of the manufacturer. The HPLC system used was comprised of a pump (Waters Alliance 2695), a loop injector (Rheodyne), and a Decade ECD that contained a glassy carbon electrode programmed to a potential of, respectively, 50 mV. The software that was used to control the produced current was Empower Pro (Waters Corporation, Milford, MA, USA). The concentrations between 1 and 1000 nM were used to generate the calibration curve. This curve was used to calculate the concentration of serotonin in each sample that was analyzed, following the recommendations of the manufacturer.

### 2.8. Statistical and Data Analysis

GraphPad Prism 8 software was used to design graphs, calculate IC_50_ values, and perform statistical analysis. The results are presented as mean ± SD for three independent experiments. Statistical comparisons between control and treatment groups, at the same time point, were performed with Student’s t-test and one-way ANOVA test. Differences were significant when *p*-value < 0.05.

## 3. Results

### 3.1. MCF-7 Growth Curve

**Figure 3:**
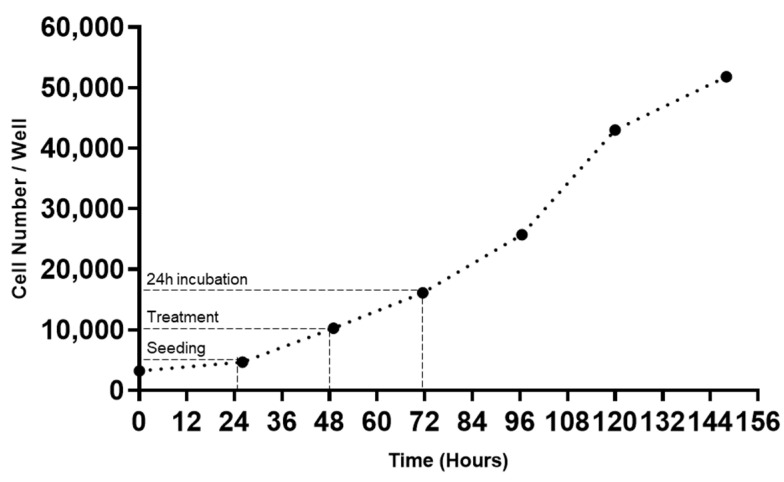
Growth curve of MCF-7 cells, obtained by staining these cells with the Hoechst 33342 dye and counting the total cell number, per well, for 148 h. Results from three independent experiments.

The maintenance of an exponential growth phase of cellular growth is important to ensure genetic/phenotypic stability, as well as cellular viability [21]. It is, therefore, important to generate a growth curve for the cell line to determine its inherent growth characteristics. Thus, to verify the growth of the MCF-7 cells used in this work, the growth curve of these cells was carried out (Figure 3). The procedure is described in the Materials and Methods section.

Analyzing the obtained curve, it is possible to conclude that, in agreement with our previous work [15], with the seeding density of 2.3 × 10^4^ cells/mL (4600 cells/well), the cells remained in the exponential growth phase during the experiments, ensuring the stability and viability of MCF-7 cells.

### 3.2. Effect of Paclitaxel on MCF-7 Cellular Viability

**Figure 4:**
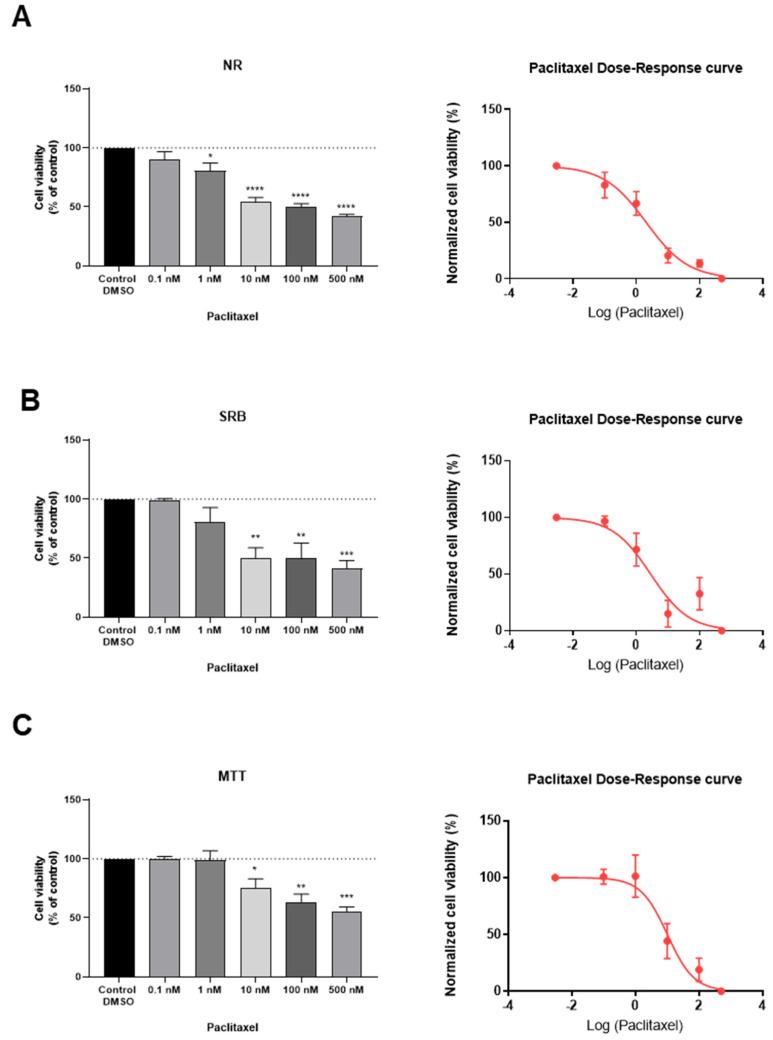
Effects of PTX on the viability of MCF-7 cells determined by (**A**) NR, (**B**) SRB, and (**C**) MTT assays. Cells were treated with increasing concentrations of PTX for 24 h and dose-response curves for each assay were obtained (right). For the construction of the dose-response curves, the data was normalized between 0% (lowest obtained value of cellular viability) and 100% (highest obtained value of cellular viability). The results are shown as the percentage of control and are expressed as the mean ± SEM of three independent experiments (*n* = 3). * Statistically significant vs. control at *p* < 0.05; ** statistically significant vs. control at *p* < 0.01; *** statistically significant vs. control at *p* < 0.001; **** statistically significant vs. control at *p* < 0.0001.

**Figure 5:**
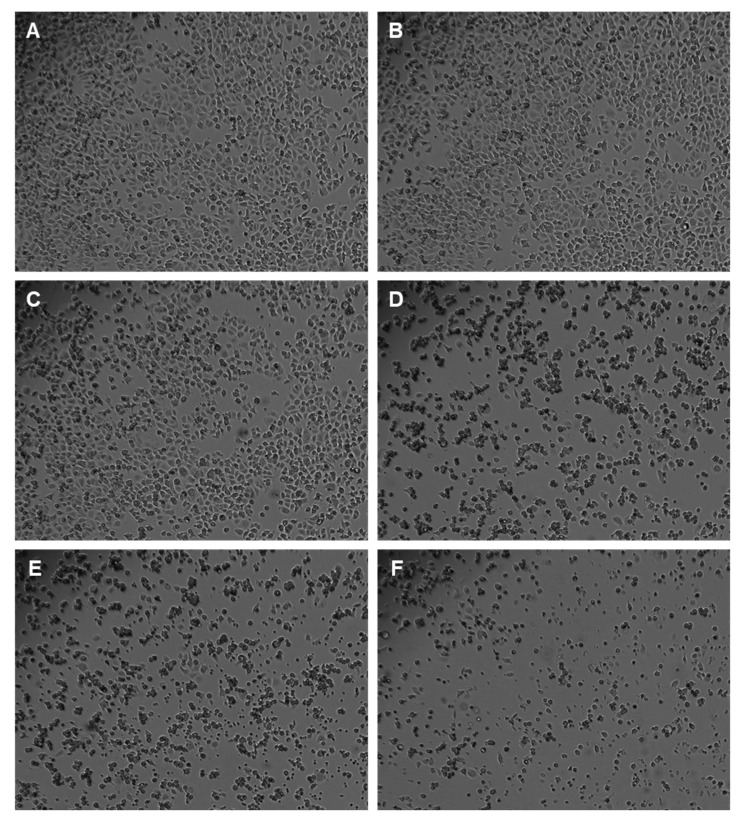
Effects of PTX on the morphology of MCF-7 cells. Cells were treated with (**A**) vehicle (DMSO), (**B**) 0.1 nM, (**C**) 1 nM, (**D**) 10 nM, (**E**) 100 nM, and (**F**) 500 nM PTX. Representative images were obtained with a high contrast brightfield objective (10×) (LionHeart FX Automated Microscope), from three independent experiments.

To assess the effect of an antineoplastic drug, clinically used in breast cancer therapy (paclitaxel, PTX) on the viability of MCF-7 cells, this drug was added to cells in concentrations ranging from 0.1 nM to 500 nM, for a period of 24 h of incubation. After this time, the percentage of viable cells was obtained using different cell viability methods, namely NR uptake assay (Figure 4A), SRB assay (Figure 4B), and MTT assay (Figure 4C), as described in the Materials and Methods section. In addition, the concentration-response curves and the respective IC_50_ values of this drug were also determined (Figure 4A–C; Table 2). The observation and morphological analysis of the cells treated with the different concentrations of this established antineoplastic agent was also carried out (Figure 5).

Our results reveal that from concentrations of 10 nM, there was a significant decrease in the cellular viability (Figure 4A–C and Figure 5D), and this response was more pronounced in both NR and SRB assays (Figure 4A,B). Additionally, with concentrations of 2.042 nM (Figure 4A), 2.850 nM (Figure 4B), and 10.12 nM (Figure 4C), there was a reduction of about 50% of the cells. These values of concentration represent the IC_50_ values of PTX, defined as the concentration showing 50% of cell growth inhibition when compared to control. For the same period of treatment with PTX (24 h), in MCF-7 cells, these values are consistent with the literature [22]. Indeed, there was an observed decrease in cellular viability, in a concentration-dependent manner. Higher concentrations of paclitaxel led to fewer and more damaged cells, as well as more rounded/smaller morphologies, typical of non-viable cells (Figure 5). Taken together, these results support the antineoplastic activity of PTX in MCF-7 breast cancer cells.

### 3.3. Effect of Isoprenaline, Propranolol, and ICI 118,551 on MCF-7 Cellular Viability

**Figure 6:**
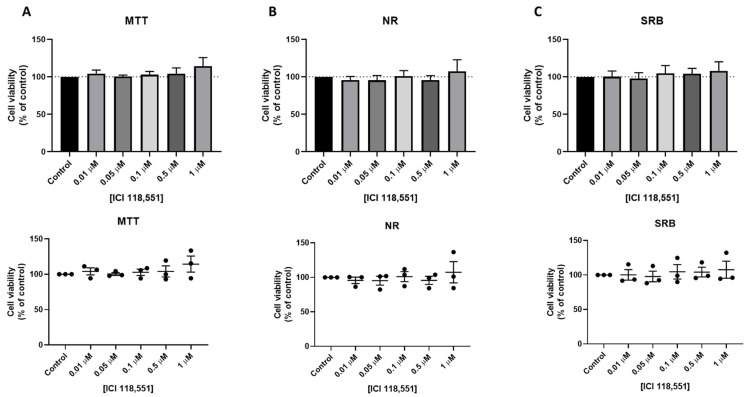
Effects of ICI 118,551 on the viability of MCF-7 cells determined by (**A**) MTT, (**B**) NR, and (**C**) SRB assays. Cells were treated with increasing concentrations of ICI 118,551 for 24 h. The results are shown as the percentage of control and are expressed as the mean ± SD of three independent experiments.

We next evaluated the effect of three β-adrenergic drugs in the viability of MCF-7 breast cancer cells. Cells were treated with increasing concentrations of ICI 118,551, propranolol, and isoprenaline for 24 h and cellular viability was evaluated by MTT, NR, and SRB assays. ICI 118,551 treatment caused an increase in MCF-7 cell viability, which can be related to increased cell proliferation, mainly in the higher concentration (1 µM) (Figure 6).

**Figure 7:**
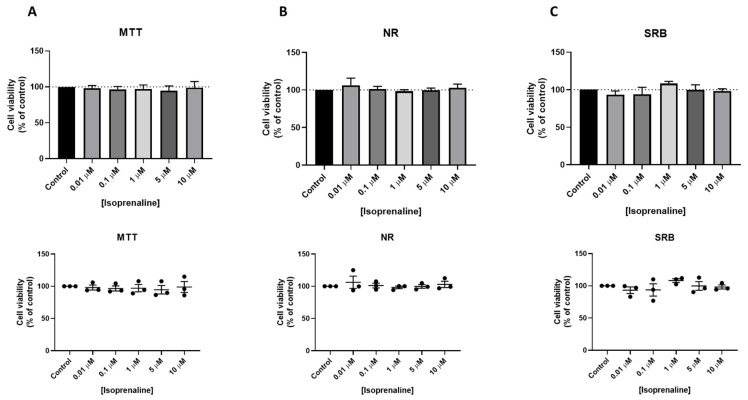
Effects of isoprenaline on the viability of MCF-7 cells determined by (**A**) MTT, (**B**) NR, and (**C**) SRB assays. Cells were treated with increasing concentrations of isoprenaline for 24 h. The results are shown as the percentage of control and are expressed as the mean ± SD of three independent experiments.

Cells treated with isoprenaline did not show any significant alterations in cell viability (Figure 7A–C).

**Figure 8:**
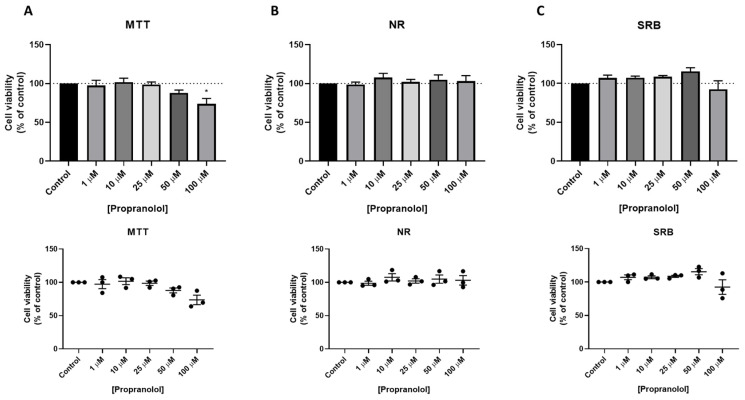
Effects of propranolol on the viability of MCF-7 cells determined by (**A**) MTT, (**B**) NR, and (**C**) SRB assays. Cells were treated with increasing concentrations of propranolol for 24 h. The results are shown as the percentage of control and are expressed as the mean ± SEM of three independent experiments (*n* = 3). * Statistically significant vs. control at *p* < 0.05.

Treatment of MCF-7 cells with propranolol resulted in a decrease of cell viability in the highest concentration (100 µM), both by MTT (Figure 8A) and SRB (Figure 8C) assays. This decrease was statistically significant by MTT assay. Results by NR assay did not demonstrate significant changes in cell viability (Figure 8B).

### 3.4. Effect of Isoprenaline, Propranolol, and ICI 118,551 on MCF-7 Serotonin Production

**Figure 9:**
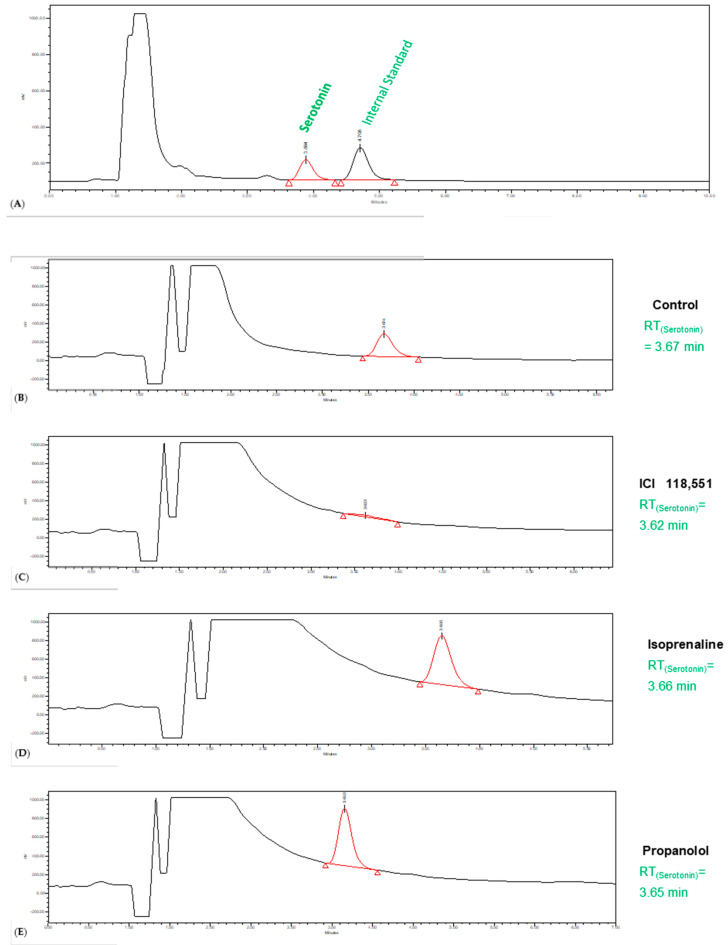
Analysis of 5-HT by HPLC-ECD. Representative chromatograms of (**A**) internal standard containing the indicated monoamine, (**B**) cell culture medium sample of untreated cells (control), (**C**) cell culture medium sample of ICI 118,551 treated cells, (**D**) cell culture medium sample of isoprenaline treated cells, and (**E**) cell culture medium sample of propranolol treated cells. Representative chromatograms were obtained in three independent experiments (*n* = 3).

Intending to verify the influence of the 24 h treatment of the cells with propranolol (100 µM), ICI 118,551 (1 µM), and isoprenaline (10 µM) in the endogenous production of 5-HT by MCF-7 cells, we proceeded to the HPLC-ECD analysis of the synthesis of this monoamine by the cells (Figure 9), enabling its quantification (Table 3). This methodology has the great benefit of enabling quick results, mainly due to short analysis times, and simple/efficient sample preparation. The entire experimental procedure is described in the Materials and Methods section.

Analyzing Figure 9 and Table 3, it is possible to notice that 5-HT was detected in all the conditions (treatment and control). However, there were clear differences between the different conditions of treatment. The drug isoprenaline promoted the most pronounced endogenous synthesis of serotonin, about 3.5-fold greater than what was obtained in the control. Nevertheless, with propranolol treatment, it was also possible to notice an increase in the synthesis of 5-HT (when compared to control), about 1.5-fold. On the other hand, treatment with the drug ICI 118,551 promoted a lower endogenous synthesis of 5-HT, about 1.1-fold less than what was observed in the control. Together, these results reveal that MCF-7 cells can produce 5-HT, and the drugs propranolol, isoprenaline, and ICI 118,551 influence this endogenous production.

## 4. Discussion

Several pieces of evidence have demonstrated the importance of neurotransmitters in the oncological context. With a particular focus on 5-HT, it is known that this monoamine is very important in several processes of cancer development, such as in its progression [1]. In this study, it was reported that MCF-7 breast cancer cells produce their own 5-HT and that this monoamine influences the viability of these cells, after the application of drugs that interact with the adrenergic system—propranolol, ICI 118,551, and isoprenaline—a period of 24 h of treatment. Here, propranolol acts as a partial agonist, whereas ICI 118,551 acts as an inverse agonist. Thus, this study suggests an interaction, in the oncological context, between β-adrenergic drugs and the production of 5-HT by MCF-7 cells. Indeed, greater cellular growth can be related to higher consumption of serotonin by the cells and, consequently, less amount of serotonin is detectable in the cell medium. It is important to note that the use of PTX, an anticancer drug clinically used in the context of breast cancer [23], can give us security in the process of connecting the results of cell viability with the results obtained by HPLC, after the β-adrenergic treatment of the cells. This is mainly because propranolol, ICI 118,551, and isoprenaline are not typically cytotoxic drugs. Indeed, these drugs do not have a defined profile regarding the cell viability response, such as PTX. Thus, with the use of a drug with an established profile in breast cancer, it was possible to discard that fluctuations regarding the cell viability results obtained with the β-adrenergic drugs were due to the methods of cell viability or due to problems at the cellular level, making the connection between 5-HT production and cellular viability more reliable.

The 24 h treatment period was chosen considering the 5-HT metabolism. This monoamine, produced from tryptophan, is metabolized to other compounds. The main and most abundant metabolite of 5-HT is 5-hydroxyindoleacetic acid (5-HIAA), typically excreted in the urine and clinically used for the diagnosis of carcinoid syndrome, through a 24 h urine sample [24,25,26]. Thus, in a period greater than 24 h, more products of serotonin metabolism (such as 5-HIAA) could be observed and not 5-HT itself, which is the focus of this article.

Focusing on the treatment of the cells with propranolol, it is known that this drug is being studied in the context of drug repurposing in cancer, highlighting breast cancer [27]. Indeed, preclinical and retrospective studies suggest that this beta-blocker may improve the prognosis of patients with early-stage breast cancer, being a safe and low-cost option [28]. Several mechanisms of action may explain the anti-tumoral effects of propranolol. For example, studies show that this drug decreases the expression of Ki-67 (pro-proliferative marker) and Bcl-2 (pro-survival marker), and increases the expression of p53 (pro-apoptotic), disrupting the progression of the cell cycle [29].

Nevertheless, there is still a lot to investigate about the anti-tumoral effects of this drug. Thus, one of the mechanisms of action that may explain this action of propranolol may also be due to its interplay with the serotonergic system. With the application of propranolol, high amounts of serotonin (when compared to control) were found in the cellular medium, which supports the hypothesis that the cells did not use this monoamine to develop their growth, an effect that was the opposite with the ICI 118,551. One of the processes that may be occurring is the antagonism of the 5-HT1A and 5-HT1B receptors (Table 1), which, being blocked, may prevent 5-HT from exerting its effects, supporting the anti-tumour mechanism of propranolol, leading to a decrease in the growth/viability of tumour cells by preventing the pro-tumoral action of 5-HT. This same effect may explain the results obtained with isoprenaline and ICI 118,551. This hypothesis highlights the importance of developing more cancer studies with β-adrenergic drugs, focusing on the study of the receptors to which they can bind, such as 5-HT receptors. Indeed, the mechanism of interaction of these drugs with the serotonergic system may be because these drugs interact with different 5-HT receptors. More studies about these processes are extremely important and relevant. These findings also provide new perspectives to consider about the study of neurotransmitters and their interplay in the oncological context.

## 5. Conclusions

**Figure 10:**
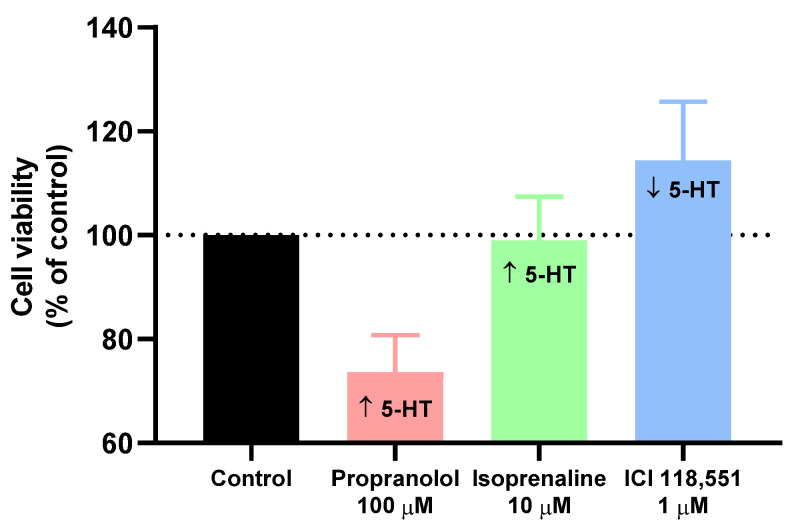
Relationship between cell viability and endogenous 5-HT values of MCF-7 treated with propranolol, isoprenaline, and ICI 118,551. Values of cell viability obtained by MTT assay. The results are shown as the percentage of control and are expressed as the mean ± SD of three independent experiments.

Our results demonstrate that 5-HT quantification is different among MCF-7 cells treated with three β-adrenergic drugs (propranolol, isoprenaline, and ICI 118,551) and that 5-HT levels in cell media correlate with cell viability determined after each treatment (Figure 10).

These results confirm that 5-HT has a relevant positive role in the growth of breast cancer cells [14], more pronounced in our study with the drug that interacts with β-adrenergic and 5-HT receptors—propranolol. We also founded that propranolol, whose treatment resulted in decreased cell viability, resulted in higher 5-HT levels in cell media, indicating fewer needs of 5-HT by MCF-7 cells. On the other hand, cells treated with ICI 118,551 that resulted in higher cell growth rates, revealed a higher consumption of 5-HT, resulting in decreasing levels of 5-HT compared to control cells in HPLC quantification. These results support that enhanced cell growth can be related to higher 5-HT consumption by the cells, resulting in decreased levels of 5-HT in cell media and indicating that 5-HT may be necessary for MCF-7 cellular growth.

## Figures and Tables

**Table 1:** Known targets of isoprenaline, propranolol, ICI 118,551, and respective chemical structure, action/pKi values on these targets. Data from [18,19,20].

Drug	Chemical Structure	Target	pKi	Action
Isoprenaline	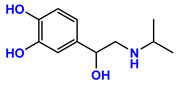	β1—Adrenoreceptor	6.6–7.0	Agonist
		β2—Adrenoreceptor	6.4	Agonist
Propranolol	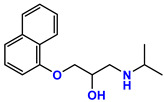	β1—Adrenoreceptor	8.6–8.8	Antagonist
		β2—Adrenoreceptor	9.1–9.5	Antagonist
		β3—Adrenoreceptor	6.3–7.2	Antagonist
		5-HT1A	7.5	Antagonist
		5-HT1B	5.38	Antagonist
ICI 118,551	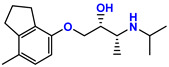	β1—Adrenoreceptor	6.6	Antagonist
		β2—Adrenoreceptor	9.2–9.5	Antagonist
		β3—Adrenoreceptor	5.8–6.6	Antagonist

**Table 2:** Obtained IC_50_ values, with respective methodologies, for the 24 h treatment of MCF-7 cells with PTX.

IC_50_ Value/nM	Methodology
2.0 (42)	NR Assay
2.8 (50)	SRB Assay
10.1 (20)	MTT Assay

**Table 3:** Variation of concentrations of 5-HT (nM) in the cellular medium of propranolol, isoprenaline, and ICI 118,551-treated MCF-7 cells, determined by HPLC-ECD.

Drug	(5-HT)/nM
Propranolol (100 µM)	+147.61 ± 6.20
Isoprenaline (10 µM)	+346.86 ± 9.12
ICI 118,551 (1 µM)	−115.81 ± 4.56

## Data Availability

Not applicable.
